# Epidemiology of *Clostridioides difficile* infections, France, 2010 to 2017

**DOI:** 10.2807/1560-7917.ES.2019.24.35.1800638

**Published:** 2019-08-29

**Authors:** Mélanie Colomb-Cotinat, Laetitia Assouvie, Julien Durand, Côme Daniau, Lucie Leon, Sylvie Maugat, Sophan Soing-Altrach, Cécile Gateau, Jeanne Couturier, Isabelle Arnaud, Pascal Astagneau, Anne Berger-Carbonne, Frédéric Barbut

**Affiliations:** 1 Santé publique France, Saint-Maurice, France; 2 These authors contributed equally and share first authorship; 3 National reference laboratory for anaerobic bacteria and *C. difficile*, St Antoine Hospital, Paris, France; 4 Regional center for prevention of healthcare associated infections, Paris, France

**Keywords:** *Clostridioides *difficile infections, PMSI, France, CDI incidence survey, epidemiology

## Abstract

**Background:**

*Clostridioides difficile* is a leading cause of healthcare-associated diarrhoea in middle and high-income countries. Up to 2018, there has been no systematic, annual surveillance for *C. difficile* infections (CDI) in France.

**Aims:**

To provide an updated overview of the epidemiology of CDI in France between 2010 and 2017 based on five different data sources.

**Methods:**

This is a descriptive study of retrospective surveillance and alerts data. Incidence of CDI cases was estimated through the CDI incidence survey (2016) and data from the French National Uniform Hospital Discharge Database (PMSI; 2010–16). Testing frequency for CDI was estimated through the CDI incidence survey and point prevalence studies on healthcare-associated infections (HAI; 2012 and 2017). The national early warning response system for HAI (HAI-EWRS, 2012–17) and National Reference Laboratory data (2012–17) were used to follow the number of severe CDI cases and/or outbreaks.

**Results:**

In 2016, CDI incidence in acute care was 3.6 cases per 10,000 patient days (PD). There was a statistically significant increase in CDI incidence between 2010 and 2016 (+ 14% annually) and testing frequency was 47.4 per 10,000 PD. The number of CDI HAI-EWRS notifications decreased between 2015 and 2017 with only a few large outbreaks reported.

**Conclusion:**

The CDI incidence estimate increased from 2010, but remained below the European average of 7 per 10,000 PD in 2014; there were fewer severe cases or clusters reported in France. The consistency between PMSI and laboratory-based estimated CDI incidence could allow for more routine monitoring of CDI incidence.

## Introduction

*Clostridium difficile*, officially renamed *Clostridioides difficile* in 2016, is responsible for 15–25% of antibiotic-associated diarrhoea cases [1,2] and is considered the leading cause of healthcare-associated diarrhoea in developed countries. *C. difficile* infections (CDI) can be severe (toxic megacolon, septic shock) and represent one of the most expensive nosocomial infections [1,3-5]. In more than 95% of CDI cases, the patient receives antibiotics just before the onset of diarrhoea [2].

The epidemiology of CDI has changed over the past 20 years with the emergence of a hypervirulent clone NAP1/027/BI implicated in large outbreaks of severe CDI worldwide with high mortality rates [6-11] in the beginning of the 2000s. Between 2006 and 2007, this clone was responsible for outbreaks of severe CDI in the north of France before spreading gradually throughout the territory [9]; these outbreaks were controlledby the end of 2007. In 2009, following the epidemic period, a national prospective, multicentric survey to assess CDI incidence and to characterise CDI strains was launched in France (ICD-Raisin study). It showed an incidence of 2.28 CDI per 10,000 patient days (PD) in acute healthcare facilities (HCF) and 1.15 per 10,000 PD in long-term facilities [12]. The European, multicentric, prospective, point prevalence study (PPS) of CDI in hospitalised patients with diarrhoea (EUCLID) study, conducted in 2014 [13], found an incidence of 3.9 per 10,000 PD in 2011-12 in France and an estimated average incidence of 7 per 10,000 PD (range 0.7–28.7) in Europe.

Up to 2018, there has been no systematic, annual surveillance for CDI in France. Since 2007, routine surveillance of CDI is based on two data sources: (i) notifications by HCF through the national healthcare-associated infections early warning and response system (HAI-EWRS) [14], which are only mandatory for severe CDI presentations and/or outbreaks. Since 2012, the HAI-EWRS has been accessible via an online application (e-SIN), (ii) microbiological data from the national reference laboratory for *C. difficile* (NRL, Paris). NRL data do not reflect the overall epidemiology of *C. difficile* in France, however, as the strains usually come from severe cases or outbreaks and sending of the strains to NRL is not mandatory.

To complement and update available data on CDI in France, a laboratory-based CDI incidence survey was conducted in 2016 through the pre-existing national multidrug-resistant bacteria surveillance programme (French acronym BMR-Raisin) [15]. The survey was offered as an optional module for laboratories within acute HCF to complete. Two national PPS of HAI and antimicrobial use were also conducted in 2012 and 2017, which included data on CDI prevalence and the number of samples tested for *C. difficile*. In addition, information on inpatient hospital stays with CDI was collected from the French national hospital stays database (French acronym PMSI), for the 2010–16 period.

The aim of this study is to provide an updated overview of the epidemiology of CDI in France based on these five different data sources: (i) the 2016 CDI incidence survey, (ii) the 2012 and 2017 PPS, (iii) PMSI data, 2010–16, (iv) HAI-EWRS notifications, 2012–17 and, (v) NRL data, 2012–17. These five data sources have never been compared to each other, and only data from the PPS have been published elsewhere.

Of the five data sources, two have the purpose of alert: (i) notification to HAI-EWRS allows for a rapid real-time alert, communication between local team and regional or national support to help implementation of control measures, (ii) microbiological data from the NRL are used to follow the epidemic clone 027 and the potential emergence of more epidemic-prone or more virulent clones.

The purpose of the other three data sources are surveillance of CDI: (i) the CDI incidence survey, launched in 2016, was useful in providing a point estimation of the CDI incidence in acute HCF, (ii) PPSs are done every 5 years in France and provide a point estimation on CDI prevalence and testing frequency in a representative sample of HCF in France, (iii) PMSI data were analysed for the first time at national level to estimate CDI incidence in acute HCF.

## Methods

### The 2016 *Clostridioides difficile* infections incidence survey

#### Source of information

Between April and June 2016, all laboratories within acute HCF in France were asked to complete an optional questionnaire including questions on: (i) algorithms used for CDI diagnosis, (ii) number of stool specimens tested for *C. difficile*, (iii) number of stool specimens that tested positive for CDI (there can be several for the same patient), and (iv) number of CDI cases and number of hospital-acquired CDI cases (HA CDI cases) for acute care wards within acute HCF. The questionnaire was in accordance with the European Centre for Disease Prevention and Control’s (ECDC) technical document for minimal *C. difficile* surveillance [16]. Participation was voluntary.

A CDI case was defined as per European Society of Clinical Microbiology and Infectious Diseases (ESCMID) recommendations [9] i.e. diarrhoeal stools or toxic megacolon and a positive laboratory assay for C. *difficile* TcdA and/or TcdB in stools or a toxin-producing *C. difficile* organism detected in stools via culture or PCR. A HA CDI case was defined as a positive sample at least 48 hours following admission, with no manifest CDI infection in the 6 months before admission. Patients hospitalised for less than 24 hours and dialysis patients were excluded. The reference algorithms for the detection of a CDI were those recommended by the ESCMID [17].

#### Analysis

The testing frequency for CDI was estimated using the ratio between the number of stools tested for *C. difficile* to the number of PD in acute HCF over the study period (data source were the annual administrative survey of HCF statistics for 2016 [18]). The incidence of stools that tested positive for CDI and the incidence of HA CDI cases per 10,000 PD was estimated using the ratio between the number of stools that tested positive for CDI or HA CDI to the number of PD in acute HCF over the study period. We also calculated the proportion of HCF performing CDI diagnosis using one of the reference algorithms.

### The 2012 and 2017 point prevalence surveys

#### Source of information

In the 2012 and 2017 PPS, data on all HAI (including CDI), treatments and risk factors were collected for all patients present on the hospital ward on the day of the survey. In 2012, the PPS was sent to all HCF in France and the participation rate was 75% (1,938/2,594). In 2017, the PPS was conducted on a representative sample of 449 HCF (stratified by region and hospital type), with 403 (91%) participating. The methodology of these PPS has been described elsewhere [19]. A patient infected with *C. difficile* was defined as a compatible clinical presentation (diarrhoeal stools or megacolon) with detection of toxins A and B in the stools or pseudomembranous colitis diagnosed after colposcopy or compatible histology at endoscopy or autopsy.

#### Analysis

The prevalence of patients infected with *C. difficile* (in 2012 and 2017) and the testing frequency (i.e. the number of stools samples tested for *C. difficile* in acute HCF per 10,000 PD, variable retrospectively collected in 2017 only, based on 2016 data) were analysed. As the 2017 PPS was only conducted on a representative sample of HCF, 95% confidence intervals (CI) were calculated for the prevalence of patients with CDI. The prevalence of patients with CDI in 2012 and 2017 was compared using multilevel models (patient, HCF and region) with a Poisson regression (adjusting for age, sex, McCabe score, immunosuppression, urinary catheter, central venous catheter, peripheral venous catheter, respiratory assistance and hospital ward). Analyses were performed with Stata version 14.1 (StataCorp, College Station, Texas (TX), United States (US)).

### The French national uniform hospital discharge database data, 2010–2016

#### Source of information

PMSI is a standardised national database describing all inpatient hospital stays [20] and is used for the production of standardised healthcare billing information and medical information concerning patients (comorbidities, age and sex). Pathologies are coded by principal diagnosis and optional associated diagnoses, by a clinician using the French version of the international classification of diseases 10th Revision (ICD-10) [21]. The ICD-10 code A04.7 ‘*C. difficile* enterocolitis’ must be used for every patient with a CDI, left to the appreciation of the physician that coded the stays. Procedures are coded using a standardised classification (*Classification Commune des Actes Médicaux* [22]). Patients can have more than one stay in the same year.

#### Analysis

The PMSI was analysed for the period 1 January 2010–31 December 2016. Stays with an ICD-10 code A04.7 for principal or associated diagnosis were extracted from the PMSI database for acute wards in acute HCF and stratified by type of facility, type of stay, age, sex and region. The variable ‘type of discharge’ was used to identify in-hospital deaths (attributability was unknown). Stays, where a colectomy was performed, were identified with the following CCAM codes: HHFA002, HHFA004, HHFA005, HHFA006, HHFA008, HHFA009, HHFA010, HHFA014, HHFA017, HHFA018, HHFA021, HHFA022, HHFA023, HHFA024, HHFA026, HHFA028, HHFA029, HHFA030 or HHFA031. To calculate incidences, the ratio between the number of cases and stays to the number of PD (source: SAE 2016) were calculated. Regional data were estimated using the variable ‘region of hospitalisation’. Incidences of stays with CDI between 2010 and 2016 were compared using Poisson regression with robust variance, adjusted for geographical region. Analyses were performed with Stata version 12 (StataCorp, College Station, TX, US).

### Healthcare-associated infections early warning and response system notifications, 2012–2017

#### Source of information

The e-SIN database contains all healthcare-associated infections (HAI) notifications that have been reported since January 2012. Notifications report data on HCF (name and location) and data on date of detection of the first case, the number of cases and death, ward of hospitalisation, the microorganism responsible for infection and any control measures. A comment box for any additional information was available.

#### Analysis

HAI-EWRS notifications received between 1 January 2012–31 December 2017 were extracted from the e-SIN database. CDI notifications were identified based on the ‘microorganism’ item. The variables ‘region’, ‘number of cases’, ‘number of deaths’, ‘infectious site’ and ‘ward’ were extracted directly from the notifications. Ribotype of the strains was extracted from comments when necessary. An outbreak was defined as a notification reporting more than one case. Proportions of CDI notifications among HAI notifications between 2012 and 2017 were compared using a variance-weighted least-squares regression. Analyses were performed with Microsoft Excel 2013.

### National reference laboratory for *Clostridioides difficile* data, 2012–2017

#### Source of information

The NRL receives strains of *C. difficile* from voluntary French laboratories for characterisation. Most of these strains are linked to cases reported in the HAI-EWRS (clusters or severe forms of CDI). These strains are characterised by multiplex PCR which detects the main virulence factors (*tcdA* and *tcdB* genes encoding toxins A and B, respectively and *cdtA* and *cdtB* genes encoding the binary toxin) and by capillary gel-based electrophoresis PCR ribotyping, as described elsewhere [23]. The strains’ susceptibility patterns to antibiotics (metronidazole, vancomycin, erythromycin, tetracycline and moxifloxacin) are determined by the disk diffusion method. Antimicrobial susceptibility testing was performed according to the 2013 French CA-SFM (Comité de l’antibiogramme de la Société Française de Microbiologie) guidelines. Stain dilution (10^8^ CFU/ml) is inoculated on Brucella Agar (Becton Dickinson) supplemented with vitamin K1 (1mg/ml) (Emprove, Merck), hemin (5 mg/L) (Applichem) and defibrinated horse blood (5%). Plates are incubated 48h at 35–37 °C, and diameters are interpreted according to the criteria for anaerobic bacteria given by the CA-SFM. *C. difficile* ATCC 700057 is used as quality control. The method was the same throughout the 5 years.

#### Analysis

Number and characteristic of strains analysed by the NRL between 2012 and 2017 were described.

### Ethical statement

Anonymous surveillance data were collected from patient charts only for the public interest mission of the French public health agency or its partners, in accordance with the French data protection authority. Analyses were only conducted on aggregated data and not on an individual level.

## Results

### The 2016 *Clostridioides difficile* infections incidence survey

In 2016, of more than 2,000 acute HCF in France, 203 participated in the CDI incidence survey, corresponding to 10% (3,056,445/30,854,819) of total PD for the same year. All hospital types (tertiary, secondary and primary) and all 17 regions in France was represented. The testing frequency for CDI was 47.4 per 10,000 PD, while the incidence of stools that tested positive for CDI was 4.7 per 10,000 PD (positivity rate of 10%). Diagnostic testing was performed using ESCMID-recommended algorithms in 65% of HCF. The incidence of CDI cases per 10,000 PD was 3.6, while the incidence of HA CDI cases was 1.9 (Table 1).

**Table 1:** Testing frequency and incidence of CDI in acute care by hospital type, France, CDI incidence survey 2016 (n = 203)

Hospital type	Number of participating HCF	Testing frequency per 10,000 PD	Stools that tested positive for CDI per 10,000 PD	CDI cases per 10,000 PD	HA CDI cases per 10,000 PD
Tertiary	12	52.8	6.1	4.7	2.7
Secondary	100	51.8	4.6	3.6	2.0
Primary	82	36.0	3.8	2.6	1.2
Specialised^a^	9	78.7	6.4	6.4	3.7
**Total**	**203**	**47.4**	**4.7**	**3.6**	**1.9**

CDI: *Clostridioides difficile* infection; HA: hospital acquired; HCF: healthcare facilities; PD: patient days.

^a^ including oncology centres only.

### The 2012 and 2017 point prevalence surveys

In 2012, of 300,330 patients included in the study, 337 had CDI (prevalence = 0.11%). In 2017, of 80,988 patients included in the study, 83 had CDI (prevalence: 0.11%; 95% CI: 0.08–0.14). After adjusting for the indicators of severity, the prevalence of patients diagnosed with CDI remained stable between 2012 and 2017 (p > 0.05).

In 2017, the mean rate of *C. difficile* testing frequency was 15.9 per 10,000 PD. Large differences were observed across the HCF categories (Table 2), with tertiary care hospitals having the highest rates. Most patients (54/83; 65%) were hospitalised in secondary and tertiary hospitals (Table 2).

**Table 2:** Mean rates of *Clostridioides difficile* testing frequency and prevalence of patients diagnosed with *C.difficile* by hospital type, France, 2012 and 2017 PPS

Hospital type	Testing frequency per 10,000 PD		Prevalence of patients diagnosed with CDI						
	PPS 2017		PPS 2012			PSS 2017			
	Rate	95% CI	Number of patients included	Number of patients diagnosed with CDI	Prevalence (%)	Number of patients included	Number of patients diagnosed with CDI	Prevalence (%)	95% CI
Tertiary	68.11	41.77–94.45	58,078	136	0.23	28,688	33	0.16	0.10–0.24
Secondary	39.82	37.03–42.61	78,810	87	0.11	21,411	21	0.09	0.06–0.15
Primary	17.78	15.24–20.32	94,568	79	0.08	17,338	16	0.10	0.06–0.16
Specialised 2^a^	58.74	NA^b^	2,267	1	0.04	978	3	0.25	0.08–0.84
Subtotal acute HCF	23.48	20.73–26.23	233,723	303	0.13	68,415	73	0.11	0,09–0,15
Specialised 1^c^	3.79	2.8–4.78	66,607	34	0.05	12,573	10	0.08	0.04–0.17
**Total**	**15.86**	**4.13–17.59**	**300,330**	**337**	**0.11**	**80,988**	**83**	**0.11**	**0.08–0.14**

CDI: *Clostridioides difficile* infection*;* CI: confidence interval; HCF: healthcare facilities; NA: not available; PD: patient days; PPS: point prevalence survey.

^a^ Including oncology centres only.

**^b^** When there is only one primary sampling unit within a stratum, there is insufficient information to compute an estimate of that stratum's variance.

^c^ Including psychiatric care, rehabilitation centres and long-term facilities.

### The French national uniform hospital discharge database data, 2010–2016

**Figure 1:**
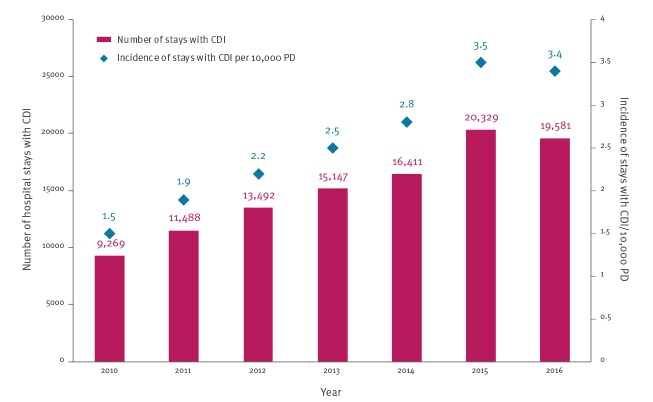
Number and incidence of hospital stays with CDI, France, PMSI data 2010–2016

CDI: *Clostridioides difficile* infection; PD: patient days; PMSI: The French national uniform hospital discharge database.

**Figure 2:**
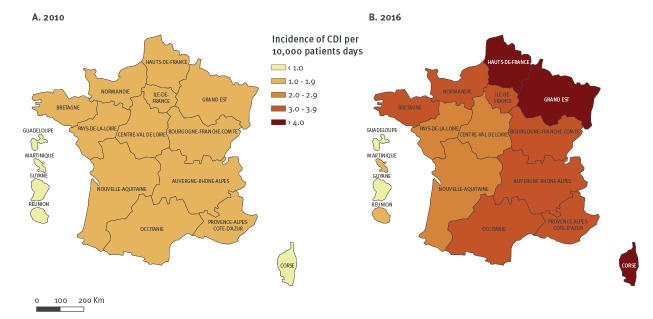
Regional incidence of CDI stays, France, PMSI data 2010 and 2016

CDI: *Clostridioides difficile* infection; PSMI: The French national uniform hospital discharge database.

Source: Geofla région 2016, Santé Publique France.

Between 2010 and 2016, 86,953 patients were hospitalised in acute HCF in France with a coded diagnosis of CDI, corresponding to 105,717 stays. A steady increase was observed from 2010 to 2015, followed by a slight decrease in 2016. The estimated incidence of stays with a CDI diagnosis significantly increased from 1.5 per 10,000 PD in 2010 to 3.4 per 10,000 PD in 2016 (+ 14% per year (95% CI: 13–16), Figure 1). This increase was observed in all French regions (Figure 2).

Of 86,953 patients hospitalised with CDI in acute HCF in France, 71,301 (82%) had only one stay, 11,304 (13%) had two and 4,348 (5%) had more than two. Patients aged 80 years or older accounted for 31,717 (36%) of patients, while those aged 14 years or less accounted for only 1,897 (2%). The sex-ratio was balanced in all age groups, except in patients aged over 80 years, where the proportion of women was higher (20,910/31,717; 66% women).

CDI was coded as the principal diagnosis in 39% (40,803/105,717) of stays. Of 64,914 stays where CDI was recorded as an associated diagnosis, the most frequent principal diagnoses were palliative care (2,281; 3.5%). In total, 3,746 different principal diagnoses were listed.

The proportion of stays where death occurred was 12% (12,614/105,717) and colectomies were performed in 1% of stays (1,250/105,717). These proportions calculated for each year are stable during the study period. These proportions were also stable when the analysis was restricted to stays where *C. difficile* infection was coded as the principal diagnosis: 2,571/40,803 (6%) patients died and 151/40,803 (0.4%) colectomies were performed.

### Healthcare-associated infections early warning and response system notifications, 2012–2017

**Figure 3:**
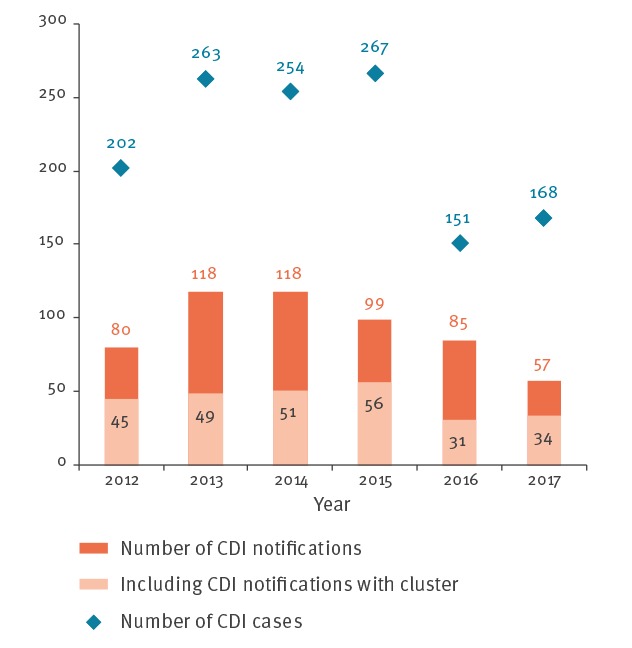
Number of HAI-EWRS notifications and cases with CDI, France, 2012–2017

CDI: *Clostridioides difficile* infection; HAI-EWRS: Healthcare-Associated Infections Early Warning and Response System.

A total of 557 notifications with *C. difficile* were received between 2012 and 2017, involving 1,305 patients and including 159 deaths (attributable or not). A decrease in the number of notifications with *C. difficile* was observed from 2015 onwards (Figure 3). The trend in the number of cases over time is more variable. Proportions of CDI notifications among all HAI notifications decreased by 1% annually 2012–17 (p < 10 ^− 5^, variance-weighted least-squares regression): CDI notifications account for 5% (80/1,551) of HAI notifications in 2012 and 2% (57/2,890) in 2017.

Of 557 notifications of CDI, 166 (30%) occurred in rehabilitation/long-term care units and 56 (10%) in geriatric units; a presumed or confirmed 027 strain of *C. difficile* was reported in 161 (29%) of the notifications.

Among all CDI notifications, 245/557 (44%) reported at least two cases. This proportion varied between 36% and 60% depending on the year. The median number of cases per episode was three (range: 2–21). A few outbreaks involving more than 10 cases were reported: two in 2012, one in 2013, one in 2014, three in 2015 and three in 2017. No large outbreaks were reported in 2016.

### National reference laboratory for *Clostridioides difficile* data, 2012–2017

Between 2012 and 2017, the frequency of PCR ribotype 027 and PCR ribotype 078/126 significantly decreased from 21.7% to 9.56% (p < 0.0001) and 12.9% to 7.49% (p = 0.02), respectively (Table 3).

**Table 3:** Number of *Clostridioides difficile* strains analysed by the NRL, by ribotype, France, 2012–2017

PCR-ribotype	Number of strains (%)											
	2012		2013		2014		2015		2016		2017	
	n	%	n	%	n	%	n	%	n	%	n	%
27	91	21.7	126	23.8	67	13.5	75	14.0	60	15.9	37	9.6
014/020/077	55	13.1	92	17.3	80	16.1	82	15.4	60	15.9	61	15.8
078/126	54	12.9	46	8.7	59	11.9	49	9.2	25	6.6	29	7.5
002	16	3.8	22	4.1	33	6.6	27	5.1	23	6.1	31	8.0
001	10	2.4	11	2.1	26	5.2	13	2.4	9	2.4	10	2.6
005	9	2.1	10	1.9	11	2.2	24	4.5	15	3.9	0	< 1
15	3	< 1	10	1.9	19	3.8	9	1.7	2	0.5	0	< 1
17	5	1.2	8	1.5	4	< 1	5	0.9	0	< 1	1	< 1
106	2	< 1	10	1.9	15	3	11	2.1	21	5.57	18	4.6
53	0	< 1	2	< 1	4	< 1	0	< 1	0	< 1	4	1.0
Other	175	41.7	193	36.4	180	36.5	239	44.7	162	43.0	197	50.9
**Total**	**420**	**100**	**530**	**100**	**498**	**100**	**534**	**100**	**377**	**100**	**387**	**100**

NRL: national reference laboratory.

In 2017, of 387 *C. difficile* toxigenic strains, 25 (6.4%) were the ‘historical’ PCR-ribotype 027, susceptible to moxifloxacin and 99 (25.7%) produced binary toxin. All strains were susceptible to metronidazole and vancomycin. The other PCR ribotypes remained relatively stable, except PCR ribotype 002 (3.8% vs 8.01%) and PCR ribotype 106 (< 1% vs 4.65%) which slowly emerged from 2012 to 2017.

## Discussion

This study has provided an updated overview of the epidemiological data available on CDI in acute HCF in France between 2010 and 2016, combining for the first time five different data sources. The CDI incidence survey, launched in 2016, was repeated in 2017 and 2018 but is time-consuming for laboratories and regional centres. The results of the 2017 and 2018 survey are not currently available (as at July 2019) and it will not be repeated after 2018. However, the CDI incidence in acute HCF in 2016 estimated using PMSI data were consistent with the incidence estimated from the CDI incidence survey conducted in the same year. A pilot study conducted in 2010 by the NRL [24] compared the sensitivity and specificity of a surveillance programme using PMSI data with laboratory-based surveillance data. It found that the PMSI data underestimated the incidence of CDI, compared with the laboratory results from the NRL (sensitivity 35.6%). This suggests the PMSI coding has improved since 2010 and that PMSI data in 2016 could closely represent the true incidence of CDI cases – opening up the possibility for a national surveillance system utilising routine monitoring of the incidence of stays with CDI (PMSI data), complemented with microbiological-based surveillance carried out by the NRL. The ICD-10 code A04.7 is specific for CDI, making easy the use of PMSI for surveillance purpose. In addition, HAI-EWRS is still needed to help monitoring alerts and repeated PPS will provide a point estimation on CDI prevalence and testing frequency in a representative sample of HCF in France.

The 2016 CDI incidence survey estimated the CDI incidence in acute HCF as 3.6 cases per 10,000 PD, which is an increase from the 2.3 cases per 10,000 PD estimated in 2009 by the ICD-Raisin study. This increase may be due to greater awareness of *C. difficile* infection among clinicians and to the development of more sensitive diagnostic tests for *C. difficile* such as PCR. In France, only diarrhoeic stool samples and patients aged 3 years and older are recommended to be tested for *C. difficile*. Test-of-cure testing after a treatment for CDI and routine screening for colonisation on non-diarrheic stools are not recommended. Only a minority of laboratories in France systematically test for *C. difficile* in all diarrhoeic stool samples, the majority only do it when requested by a physician. Therefore, the testing frequency depends on a physician’s awareness for *C. difficile*. CDI surveillance in England indicates that more than 75% of cases occur in patients aged 64 years and older [25]. Therefore, as the population is getting older and at a greater risk of CDI, an increase in incidence of CDI is possible [26].

Despite the increase of CDI in France (2010–16), the incidence of CDI in 2016 remains below the European average in 2014 (7/10,000 PD) [13], which could be explained by differences in CDI testing policies. The CDI incidence survey has estimated this *C. difficile* testing frequency at 47.4 stools tested per 10,000 PD in acute HCF in 2016, with differences regarding hospital type, while it was estimated at 62–69 stools tested per 10,000 PD on average in Europe [13]. This suggests that the proportion of stools tested for *C. difficile* is lower in France than in other European countries. The testing frequency estimated from PPS data were slightly different (23.48 stools tested/10,000 PD) and may be due to differences in participating acute HCF. In addition, data for the PPS also include long-term wards within acute HCF (lower testing frequency) whereas the CDI-incidence survey excluded data from long-term wards. Further, participation in the CDI-incidence survey was voluntary so participating HCF may have been more aware of CDI as a result of outbreaks; this potential self-selection bias could explain the higher testing frequency, compared to the testing frequency estimated by the 2017 PPS, done on a representative sample of HCF.

In parallel, the number of CDI HAI-EWRS notifications reached a peak in 2013-14 (118 notifications/year) before decreasing from 2015 onwards. There was also a decrease in the number of 027 strains identified at the NRL. The proportion of HAI-EWRS notifications involving 027 strains varied widely from year to year, but this proportion also took into account possible 027 strains not confirmed by the NRL. In addition, some laboratories use the GeneXpert method, which does not have high specificity for the 027 strain leading to an overestimation. NRL microbiology data show the emergence of PCR ribotype 002 and PCR ribotype 106 from 2012 to 2017. PCR ribotype 002 has been shown endemic in some nursing homes in Hong-Kong [27] and PCR ribotype 106 has been responsible for outbreaks in vascular surgery [28].

The low number of CDI outbreak notifications in France is less worrying than in other countries such as Germany, where the epidemic clone accounts for 40% of *C. difficile* strains [29]. The prevention and control of CDI are based primarily on appropriate microbiological testing practices, antibiotics stewardship policy and prevention of cross-transmission by implementing contact precaution [30,31].

According to the PMSI data, the proportion of severe forms (i.e. leading to death or colectomy) remained stable over time, but we do not know whether death is attributable to CDI. Two studies have already shown excess mortality related to CDI compared with age-matched and comorbidity-matched patients who did not have CDI [4,32].

The majority of CDI cases reported in our survey are HA, which is not surprising as the survey targeted only hospitalised patients and *C. difficile* is one of the most common HA pathogen [33]. For example, the 2016 European epidemiological report of CDI [34] found that HA CDI made up 74.6% of cases. The increase observed in CDI-related hospital stays differed between regions in France. Regional incidence disparities should be studied in the future and maybe partly explained by disparities in healthcare organisation, especially in overseas regions (HCF activities, specialities etc.). Incidence of CDI-related hospital stays differed with the age of the patient. It would be interesting to estimate incidence in different age groups, and that will be the subject of further work.

A limitation of our study is the difference between CDI definitions between the data sources: (i) in the CDI incidence survey, definition is based on clinical elements and microbiological confirmation as recommended per the ESCMID, (ii) in the PSSs, definition is based on clinical or histological elements +/ − detection of toxins in the stools, (iii) in the PMSI and HAI-EWRS, CDI case definition is decided by the physician coding the stays or making the notification, but should normally match the ESCMID definition as recommended in France.

### Conclusion

This study which combines five different data sources on CDI epidemiology in France for the first time shows that despite an increase of CDI incidence between 2010 and 2016, the incidence of CDI cases in France in 2016 remains below the European average. There is a low number of CDI outbreak notifications and there is a decrease in the number of 027 strains analysed by the NRL. Surveillance and alert for CDI remains however essential and thanks to this study, we have opened up the possibility for a national surveillance system utilising routine monitoring of the incidence of stays with CDI (PMSI data), complemented with microbiological-based surveillance carried out by the NRL. In addition, further studies will be needed to estimate incidence in different age groups and to explore the difference in regional incidences.
